# Anemia as a Part of the Triple Burden Among Children Under-Five with Stunting and Tuberculosis in Bandung, Indonesia

**DOI:** 10.3390/children12121620

**Published:** 2025-11-28

**Authors:** Susi Susanah, Delita Prihatni, Rini Rossanti, Safira Satyani Lutfia, Fadhila Novianti, Fedri Ruluwedrata Rinawan, Diah Asri Wulandari, Muhammad Akbar Tirtosudiro, Citra Cesilia, Sri Sudarwati, Cissy Rachiana Sudjana Prawira, Heda Melinda Nataprawira

**Affiliations:** 1 Department of Child Health, Dr. Hasan Sadikin General Hospital/Faculty of Medicine, Universitas Padjadjaran, Bandung 40161, Indonesia; rini.rossanti@unpad.ac.id (R.R.); safira16007@mail.unpad.ac.id (S.S.L.); fadhila16003@mail.unpad.ac.id (F.N.); diah.asri@unpad.ac.id (D.A.W.); akbar.tirtosudiro@unpad.ac.id (M.A.T.); sri.sudarwati@unpad.ac.id (S.S.); cbkarta@gmail.com (C.R.S.P.); heda.melinda@unpad.ac.id (H.M.N.); 2 Department of Clinical Pathology, Dr. Hasan Sadikin General Hospital/Faculty of Medicine, Universitas Padjadjaran, Bandung 40161, Indonesia; delita.prihatni@unpad.ac.id; 3 Department of Public Health, Faculty of Medicine, Universitas Padjadjaran, Sumedang 45363, Indonesia; f.rinawan@unpad.ac.id; 4 Department of Child Health, Faculty of Medicine, University of Riau, Pekanbaru 28133, Indonesia; citracesilia@lecturer.unri.ac.id

**Keywords:** anemia, children under five, stunting, triple burden, tuberculosis

## Abstract

**Highlights:**

**What are the main findings?**

• Anemia is highly prevalent among children under five in Indonesia, either as a single condition or in coexistence with other morbidities such as stunting and tuberculosis. These health issues represent critical public health challenges, as this triple burden can affect their quality of growth and development in the future.

**What is the implication of the main finding?**

• This finding reinforces the urgency of implementing a mitigating screening strategy for this preventable syndemic as early as possible through an integrated and comprehensive public health service to prevent long-term consequences in later life.

**Abstract:**

**Background/Objectives**: Children with stunting are at risk of immune function disruption and micronutrient deficiencies, leading to nutritional anemia and susceptibility to infection. This study determined the prevalence and etiology of anemia in children under five with stunting and tuberculosis (TB) and analyzed the associated factors. **Methods**: A cross-sectional study was conducted among children under five with stunting from 30 May to 13 June 2022. Participants were selected via the proportionate stratified random sampling of 74 community health centers in Bandung City, Indonesia. An anthropometric measurement was performed to determine stunting and conduct TB diagnosis, and hematology tests were performed to elaborate the anemia profiles. **Results**: In total, 138 participants were included, among which 80 (58.0%) had TB and 57 (41.3%) had anemia, mostly caused by iron deficiency anemia (IDA)—38/57 (66.7%). The coexistence of anemia in children with stunting and TB was present in 33 (23.9%) and it was associated with nutritional status (weight-for-length), *p* = 0.026. **Conclusions**: Anemia, as a part of the triple burden among children under five with stunting and TB was highly prevalent, mostly due to IDA; in this study, only nutritional status was associated with the triple burden.

## 1. Introduction

Stunting is a catastrophic condition caused by chronic poor nutrition in the first 1000 days of life, affecting 22.3% (148.1 million) of children under five in 2022 [1]. Children in Asia and Africa comprised 52% and 43% of this global population, respectively [1]. Stunting persists as a substantial global problem [1]. The World Health Assembly aims to reduce the number of children with stunting to 89 million by 2030; however, only a few countries are advancing toward these targets [1]. In 2022, the prevalence of stunting in Indonesia was 21.6%, and we committed to reducing the prevalence to 14% in 2024 [2]. Moreover, this target is still challenging because the prevalence was still 21.5% in 2023, based on the Indonesia Health Survey. Children with stunting have a higher risk of infection-induced morbidity and mortality [3]. Stunting is often accompanied by immune system disruption and susceptibility to acute and chronic infections, particularly respiratory tract infections and diarrhea [4]. A vicious cycle that results in growth abnormalities is created when infections exacerbate undernutrition [5].

*Mycobacterium tuberculosis* is the most frequent cause of mortality, estimated as affecting one-third of the population [6]. The number of patients with TB continually increases each year, affecting approximately 10.8 million people globally by 2023 [7]. Approximately 1.3 million cases (12%) were children and young adolescents aged 0–14 years [7], most from Southeast Asia (45%), Africa (24%) and the Western Pacific (17%) [7]. Indonesia is ranked as the country with the second highest number of TB incident cases worldwide after India [7]. According to the Indonesia Health Profile 2021, West Java had the highest prevalence of children and adolescents with TB, accounting for 27.2% of all TB cases in individuals aged 0–14 years [8]. Nataprawira et al. reported that of 169 children under five with stunting in Bandung City, West Java, Indonesia, 101 (59.76%) and 4 (2.37%) were diagnosed with pulmonary and latent TBs, respectively [9].

Anemia remains a critical health problem, particularly in developing countries. The prevalence of anemia has decreased steadily; however, the global anemia prevalence is still high, at 24.3%, affecting approximately 1.92 billion people worldwide in 2021 [10,11]. In Indonesia, ranked fourth in term of size of population, 23.7% of the population has anemia, approximately 26.8% and 38.5% of which are 5–15- and <5-year-old children, respectively [12,13]. Anemia is associated with high morbidity and mortality, especially in children of preschool age [14]. Oktarina et al. conducted a systematic review and meta-analysis, and demonstrated that children age 0–12 years with stunting are 1.31–6.79 times more likely to develop iron deficiency anemia (IDA) [15]. Meanwhile, Susanah et al. reported that 40.7% of their study sample experienced anemia, with IDA being the cause in 87.3% cases, and the prevalence of the co-occurrence of anemia and stunting (CAS) was 21.1% [16].

Moreover, these three conditions often coexist in a vicious cycle, of which the primary initiating factor remains uncertain. It is unclear whether chronic malnutrition leads to stunting, infection, and subsequent anemia, or whether chronic infections such as TB initiate the cascade and drive stunting and anemia through prolonged inflammation and nutrient depletion. This bidirectional and overlapping interplay complicates the initial identification of a trigger.

To the best our knowledge, despite the high prevalence of this triple burden in children under five, studies remain limited. Therefore, this study aimed to determine the prevalence and etiology of anemia in children under five with stunting and TB, as well as to analyze the associated factors.

## 2. Materials and Methods

### 2.1. Study Design, Setting and Participants

**Figure 1:**
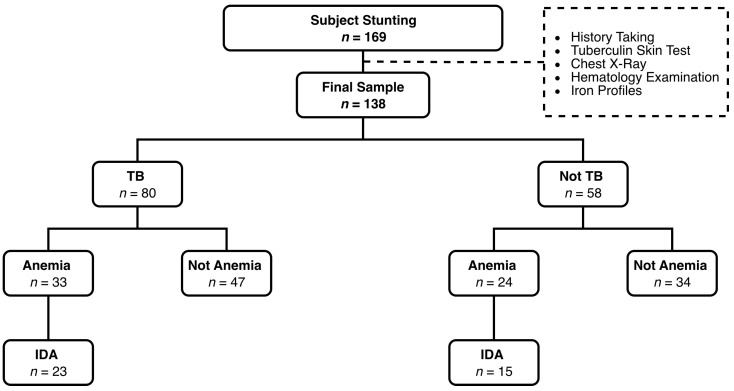
Sample selection.

This study is a part of Nataprawira et al.’s study, which investigated the TB status among children under five with stunting. A community-based cross-sectional study was conducted in 74 community health centers in Bandung City, Indonesia, involving 7568 children under five who were initially identified as having a problematic nutritional status by healthcare providers (medical doctors, midwives, and nurses). Children aged <5 years were included. A sample of 174 children under five was selected by stratified random sampling from the population frame, but 5 were excluded, leaving 169 subjects. Subsequently, the nutritional status was reassessed by the research team (pediatricians) between 30 May 2022 and 14 June 2022 in order to confirm stunting based on anthropometric measurements. Simultaneously, a tuberculin skin test (TST) and chest X-ray were performed to assess the TB status, and hematology tests were performed to screen for anemia and iron status. Children with incomplete laboratory results were excluded. A total of 138 children under five were included in the final dataset (Figure 1). The selected paired children and their mothers were invited to participate in this study. Information regarding the study was explained to the mothers, and written informed consent forms were obtained. This study was conducted in accordance with the ethical standards of the Declaration of Helsinki and was approved by the Ethical Review Board of the Faculty of Medicine, Universitas Padjadjaran (approval number: 350/UN6.KEP/EC/2022) dated 7 April 2022.

### 2.2. Data Collection and Diagnostic Criteria

As field researchers, the medical doctors were trained in standardized methods for guided interviews and physical examination. The following data were requested: age, sex, birth weight, duration of breastfeeding, history of TB contact and close contact with patients diagnosed with TB, source of drinking water, maternal height, parental education, parental occupation, and household income. Birth weight was categorized as normal if ≥2500 g and low birth weight (LBW) if <2500 g. Exclusive breastfeeding was determined if the baby was breastfed for at least 6 months. The classification of parental education was based on whether or not the participants finished each level. The household income was calculated as combined parental income 148 and recoded based on the Bandung City regional minimum wage standard cut off for categorical analysis.

The anthropometric measurement for children <2 years old used Elitech DIGIT-ONE BABYÒ/TD 05219B3340 (accredited by 81 the Ministry of Health of the Republic of Indonesia, Number 10901410291) and Elitech DIGIT-PROÒ/TD 09219B3781 was used for ≥2-year-old children. Participants were diagnosed as stunted if the anthropometric measurement indicated body length or height were less than −2SD and severely stunted if the anthropometric measurement indicated body length or height were less than −3SD based on sex and age, according to the World Health Organization growth standard charts. A child was diagnosed as wasted if the weight-for-length/height was less than or equal to −2SD/−3SDs of the median or severely wasted if the weight-for-length/height was less than −3SDs of the median [17].

Tuberculosis infection was defined as a positive TST with an induration diameter (48–72 h) of >5 mm in children with severe wasting and >10 mm in those without it, with or without clinical and/or radiological signs associated with TB. Independent pediatric respirology consultants (HMN, SS, and DAW) were recruited to assess the status of TB [9].

A venous blood sample from 169 participants, including a routine 22-parameter hematology test consisting of Hb, erythrocyte, MCV, and MCH, was obtained using Sysmex XS-800i^®^(Sysmex Corporation, Kobe City, Japan). An iron profiles test comprising serum iron (SI), total iron-binding capacity (TIBC), transferrin saturation (TS), and serum ferritin (SF) level was performed using Dimension EXL 200^®^, serial number DR270310 (Siemens Healthineers, Erlangen, Germany). Anemia was defined according to an Hb level <11 g/dL; IDA was defined as microcytic hypochromic anemia (MCV <80 fL and MCH <27 pg), and the iron profile was determined as low SI level (<50 µg/dL), increased TIBC (>450 µg/dL), low TS (<16%), and low SF level (<12 ng/mL) [18].

We differentiated hypochromic microcytic anemia conditions using the parameters as below (Table 1).

### 2.3. Statistical Analysis

The Shapiro–Wilk test was used to assess for normality in the continuous data. Data with normal distribution were presented as mean  ±  standard deviation or as median (interquartile range [IQR]) for non-normally distributed data. The categorical data were presented as frequencies and percentages and evaluated between the categorical groups. The chi-square test and Fisher’s exact test were used to determine the relevance of these differences. When the chi-square test and fisher exact test criteria were not met, the Kolmogorov–Smirnov test was performed. The unpaired-t test analyzed the independent numerical variables. Multivariate analysis was conducted to identify associated factors. The results of the multivariate analysis are expressed as Odds Ratio (OR) and 95% confidence intervals (95% CI). Statistical analysis was performed using IBM SPSS Statistics Version 30.0.

## 3. Results

### 3.1. Research Subjects

In total, 138 participants were included, with a mean age of 34.4 ± 13.7 months. The majority were female (56.5%) and the mean of birth weight was 2679.8 ± 529.9 g with LBW (26.8%). Growth impairment was dominated by severe stunting (65.2%). Based on weight-for-age, children were either underweight (49.3%) or severely underweight (27.5%). Table 2 presents the other demographic characteristics of the participants. 

**Table 2:** Characteristics of the subjects.

Characteristics	*n* = 138
Age (months) mean: ± SD	31.1 ± 12.6
Sex, *n* (%)	
Female	78 (56.5%)
Male	60 (43.5%)
Stunting status, *n* (%)	
Stunting	48 (34.8%)
Severe stunting	90 (65.2%)
Birth weight (g), Mean ± SD (*n* = 134)	2679.8 ± 529.9
Birth weight category, *n* (%)	
<2500 (g)	37 (26.8%)
≥2500 (g)	101 (73.2%)
Weight-for-age, *n* (%)	
Normal	32 (23.2%)
Underweight	68 (49.3%)
Severe underweight	38 (27.5%)
Weight-for-length, *n* (%)	
Normal	121 (87.7%)
Overweight	2 (1.4%)
Wasted	12 (8.7%)
Severe wasted	3 (2.2%)
History of TB disease, *n* (%)	1 (0.7%)
BCG immunization, *n* (%)	127 (92.0%)
BCG scar, *n* (%)	110 (79.7%)
History of TB contact, *n* (%)	8 (5.8%)
Exclusive breastfeeding, *n* (%)	118 (85.5%)
Main drinking water source, *n* (%)	
Bottled water	118 (85.5%)
Boiled tap water	12 (8.7%)
Boiled well water	8 (5.8%)
Father’s age (years), Mean ± SD (*n* = 127)	38.9 ± 7.2
Mother’s age (years), Mean ± SD (*n* = 133)	36.1 ± 7.9
Father’s education, *n* (%)	
Primary school	31 (22.5%)
Junior high school	37 (26.8%)
Senior high school	64 (46.4%)
University	6 (4.3%)
Mother’s education, *n* (%)	
Primary school	28 (20.3%)
Junior high school	41 (29.7%)
Senior high school	58 (42.0%)
University	11 (8.0%)
Father’s occupation, *n* (%)	
Government employee	42 (30.4%)
Entrepreneur	20 (14.5%)
Laborer	59 (42.8%)
Others	17 (12.3%)
Mother’s occupation, *n* (%)	
Housewife	130 (94.2%)
Worker	8 (5.8%)
Mother’s height (cm), Mean ± SD (*n* = 128)	148.4 ± 5.4
Mother’s height category, *n* (%)	
<150 cm	74/128 (57.8%)
≥150 cm	54/128 (42.2%)
Monthly household income, *n* (%)	
<IDR 2,500,000	102 (73.9%)
≥IDR 2,500,000	36 (26.1%)

Of 57 participants with anemia, most (38/57 = 66.7%) had IDA. Iron deficiency was observed in 27 participants without anemia and some showed impending anemia with hemoglobin levels near the anemia threshold as shown at the Table 3. No participants were identified with ACD alone, whereas 12 presented with IDA and ACD coexistence, 1 with concurrent IDA and hemoglobinopathy/thalassemia minor/trait, and 1 with hemoglobinopathy/thalassemia minor/trait alone, requiring further confirmation through hemoglobin analysis examination. 

### 3.2. Factors Associated with Triple Burden

**Figure 2:**
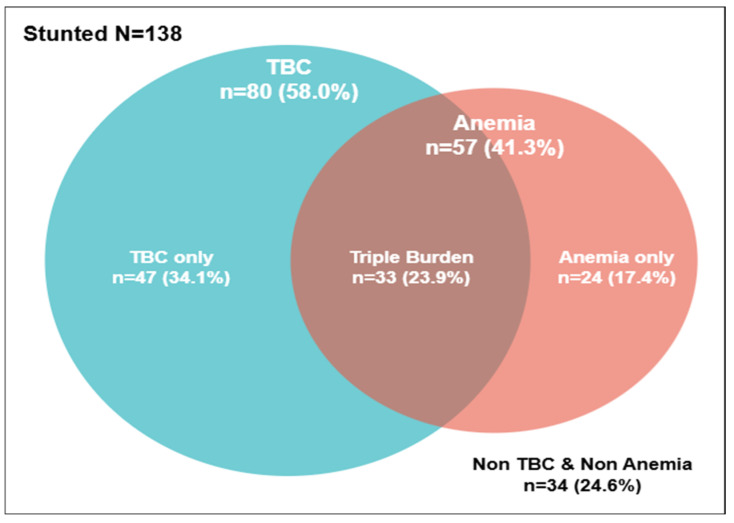
Distribution of triple burden.

In total, 57 (41.3%) participants suffered from anemia, 80 (58.0%) from TB, and 33 (23.9%) were defined as triple burden cases (Figure 2). Table 4 shows the associated factor for triple burden was weight-for-length.

The results of the multivariate logistic regression analysis of factors that have a significant association with triple burden are presented in Table 5. Weight-for-length was a predictor factor associated with triple burden in this study (*p* = 0.026).

## 4. Discussion

The high burden of devastating consequences in the later life of a child is an underlying reason for the urgent need to address stunting [21]. Stunting and anemia remain critical challenges in global child health, despite the gradual improvements in recent years. Although the prevalence of stunting and anemia among children is steadily decreasing, the incidence of TB has not shown a comparable reduction significantly, allowing these conditions to persist as substantial health concerns worldwide [1]. Malnutrition poses a notable risk factor for TB infection. There has been a twofold increase in the stunting-related risk of TB [22,23]. However, anemia may result in immunosuppression and increased TB susceptibility [24]. TB and anemia could increase each other’s prevalence [24,25]. Moreover, due to chronic anemia, it is estimated that a large number of children experience chronic malnutrition, which results in a high prevalence of stunting, and vice versa [26].

This study investigated the triple burden of anemia in children under five with stunting and TB in Bandung City, Indonesia. Previous studies have elaborated the association between stunting and anemia, stunting and TB, and TB and anemia, demonstrating that both stunting and TB may contribute to the development of anemia. Conversely, anemia may also contribute to the development of stunting [15,16,24,26].

In this study, the subject profiles showed: (1) the degree of growth impairment was dominated by severe stunting with a high number of underweight individuals, indicating a widespread chronic nutritional deficiency; (2) a significant proportion of LBW, reflecting an important risk factor for suboptimal growth and early-life morbidity; (3) a high rate of maternal short stature; and (4) extensive socioeconomic deprivation. These four factors represent key determinants of anemia—through inadequate micronutrient intake and recurrent infections—while simultaneously increasing susceptibility to TB. Within the framework of the “triple burden”, these baseline findings support the hypothesis that anemia among stunted children is likely driven by a combination of iron deficiency (due to poor diet quality), chronic inflammation, and infectious exposure. Basic health practices, such as exclusive breastfeeding and BCG immunization, were generally adequate but insufficient to counteract the underlying structural determinants of malnutrition and poverty.

Figure 2 shows a substantial overlap among these conditions, suggesting that TB infection and anemia frequently co-occur in stunted children, potentially through biological and social mechanisms, such as chronic inflammation and hepcidin-mediated iron sequestration, inadequate micronutrient intake, and environmental vulnerability factors such as poverty, household crowding, and TB exposure. The coexistence of TB and anemia in stunted children poses a compounded risk for impaired growth and development, increases susceptibility to recurrent morbidity, and delays nutritional recovery.

Children with stunting and TB are particularly vulnerable to various type of anemia. Nutritional anemia, predominantly caused by ID, frequently coexists with ACD. In addition, some cases presented with iron depletion and ID without anemia, while others suggested impending anemia. Iron plays a vital role in every tissue of a child’s growing body [27]. Iron deficiency, whether or not it is accompanied by anemia, can impair immune system performance and brain development during the first 1000 days of life, potentially resulting in delayed growth and developmental progress [28]. While anemia may be treatable, the neurocognitive impairments associated with iron deficiency may become irreversible [28]. Therefore, the early identification of the underlying cause of anemia is essential to prevent long-term adverse effects. Beyond ID, children with stunted development may experience other nutritional anemias, such as folate deficiency and vitamin B12 deficiency, further contributing to alterations in erythrocyte indices. IDA and ACD frequently present as microcytic hypochromic anemia and intervene in iron homeostasis, leading to heterogeneous iron profiles possibly complicating the interpretation of hematological assessments. In addition, Indonesia, being part of the thalassemia belt, has a relatively high prevalence of thalassemia traits, which manifest as microcytic hypochromic anemia and should be considered in the differential diagnosis of thalassemia.

Furthermore, uncertainties persist concerning the background mechanisms and clinical implications of overlapping disruptions in iron homeostasis across several forms of hypochromic microcytic anemia. Serum ferritin, a commonly used biomarker for iron status, also acts as an acute-phase reactant, leading to variable levels in conditions such as IDA, ACD, and hemoglobinopathy/thalassemia.

This study analyzed the etiology of anemia among children with stunting and TB through CBC parameters and iron profile assessment. Hemoglobin level and red cell indices, particularly MCV and MCH, were evaluated and integrated with the iron profile characteristics to achieve a more comprehensive interpretation of the results. Ideally, some biomarkers, such as hepcidin, soluble transferrin receptor and C-reactive protein, should be measured to provide a comprehensive assessment of iron metabolism and inflammatory status for precise diagnosis. In addition, hemoglobin electrophoresis is required to confirm thalassemia diagnosis. Nevertheless, these examinations were not performed owing to cost constraints.

The data are cross-sectional, and therefore, the causal relation between TB and anemia cannot be fully established. Moreover, defining anemia based on hemoglobin and iron status indicators has limited sensitivity and specificity in the presence of infection or inflammation. When determining the etiology of anemia, specific timing considerations must be made to guide appropriate management, particularly in children with this triple burden. For instance, the management of stunting and TB should be implemented simultaneously, whereas iron replacement therapy should be delivered at an optimal time [29]. In contrast, if the anemia is identified as ACD, the primary approach involves managing the underlying conditions, as anemia associated with infection often improves once the infection is adequately treated [30].

However, we recognize that determining which condition initially developed in a child may be challenging. This triple burden condition is a preventable disease; whether it occurs singly and more so if two or all three conditions occur concurrently, it constitutes a real syndemic public health problem that should be handled in a comprehensive and integrated manner. These findings emphasize the importance of implementing integrated screening strategies in addressing this condition. Stunted children diagnosed with TB should be screened for anemia, while stunted children presenting with anemia should be assessed for possible TB exposure. An integrated approach is crucial to breaking the cycle of infection, inflammation, and micronutrient deficiency, while simultaneously enhancing the efficiency of service delivery at the primary healthcare level, and this may generate greater health impacts.

## 5. Conclusions

Anemia was found to be highly prevalent in children under five with stunting and TB, mostly due to IDA; nutritional status was identified as a factor associated with the triple burden. This syndemic is a closely interrelated and preventable community health burden that should be detected early and handled in an integrated manner. In clinical practice, health providers should carry out the initial assessment of children under five comprehensively, with anthropometry measurements, TB and other infectious disease screening, and initial routine hematology tests, if indicated. As the triple burden is a public health problem, this finding emphasizes the importance of implementing integrated screening strategies in community health centers.

## Figures and Tables

**Table 1:** The differential diagnosis of iron profiles in anemia microcytosis [19,20].

Variables	IDA	ACD	Combination IDA and ACD	Minor Thalassemia
Serum Iron	Reduced	Reduced	Reduced	Normal
TIBC	Elevated	Reduced	Normal/reduced	Normal
Transferrin	Elevated	Reduced/normal	Reduced	Normal
Transferrin saturation	Reduced	Reduced	Reduced	Normal
Ferritin serum	Reduced	Normal/elevated	Reduced/normal	Normal

**Table 3:** Distribution of laboratory results.

Variables	Median (IQR)
Hemoglobin (g/dL)	9.2 (6.4–11.9)
Hematocrit (%)	34 (31.5–35.9)
Erythrocytes (×10^6^/μL)	4.49 (4.22–4.88)
MCV (fL)	75.8 (72.6–78.5)
MCH (pg)	25 (23.2–26)
MCHC (g/dL)	32.8 (32.1–33.4)
Serum iron (μg/dL)	51 (34–74.5)
TIBC (μg/dL)	347 (310–390)
Transferrin saturation (%)	15.3 (8.8–21.3)
Ferritin serum (μg/L)	19.2 (10.7–36.2)
Mentzer index	16.6 (15–18.6)
Shine and Lal index	1454 (1230–1609)

**Table 4:** Univariate analysis of the factors that were associated with triple burden.

Characteristics	Triple Burden (*n* = 33)	Non-Triple Burden (*n* = 105)	*p*
Subjects (*n* = 138)			
Age (months) mean: ± SD	31.1 ± 12.6	35.3 ± 13.8	0.126 ^a^
Sex, *n* (%)			
Female	19 (57.6%)	59 (56.2%)	0.889 ^b^
Male	14 (42.4%)	46 (43.8%)	
Stunting status, *n* (%)			
Stunting	11 (33.3%)	37 (35.2%)	0.841 ^b^
Severe stunting	22 (66.7%)	68 (64.8%)	
Birth weight (g), Mean ± SD	2627 ± 500	2697 ± 541	0.514 ^a^
Birth weight category, *n* (%)			
<2500 (g)	9 (27.3%)	28 (26.7%)	0.945 ^b^
≥2500 (g)	24 (72.7%)	77 (73.3%)	
Weight-for-age, *n* (%)			
Norml	11 (33.3%)	21 (20.0%)	0.113 ^b^
Underweight	22 (66.7%)	84 (80.0%)	
Weight-for-length, *n* (%)			
Normal	26 (78.8%)	97 (92.4%)	0.029 ^b^*
Wasted	7 (21.2%)	8 (7.6%)	
History of TB disease, *n* (%)	0 (*0*.0%)	1 (1.0%)	1.000 ^c^
BCG immunization, *n* (%)	31 (93.9%)	96 (91.4%)	1.000 ^c^
BCG scar, *n* (%)	27 (81.8%)	83 (79.0%)	0.730 ^c^
History of TB contact, *n* (%)	2 (*6*.1%)	6 (5.7%)	1.000 ^c^
Exclusive breastfeeding, *n* (%)	30 (90.9%)	88 (83.8%)	0.404 ^c^
Main drinking water source, *n* (%)			
Bottled water	31 (93.9%)	87 (82.9%)	0.200 ^b^
Boiled tap water	2 (6.1%)	10 (9.5%)	
Boiled well water	0 (0.0%)	8 (7.6%)	
Father’s age (years), Mean ± SD	39.0 ± 6.7	38.9 ± 7.4	0.967 ^a^
Mother’s age (years), Mean ± SD	36.8 ± 7.3	35.9 ± 8.1	0.562 ^a^
Father’s education			0.769
Primary school	6 (21.4)	22 (21.5)	
Junior high school	9 (32.2)	27 (26.5)	
Senior high school	11 (39.3)	48 (47.1)	
University	2 (7.1)	4 (3.9)	
Mother’s education			0.750
Primary school	4 (14.3)	23 (22.5)	
Junior high school	9 (32.1)	27 (26.5)	
Senior high school	14 (50.0)	43 (42.2)	
University	1 (3.6)	9 (8.8)	
Father’s occupation, *n* (%)			
Government employee	13 (39.4%)	29 (27.6%)	0.585 ^b^
Entrepreneur	5 (15.2%)	15 (14.3%)	
Laborer	12 (36.4%)	47 (44.8%)	
Others	3 (9.0%)	14 (13.3%)	
Mother’s occupation, *n* (%)			
Housewife	31 (93.9%)	99 (94.3%)	1.000 ^c^
Worker	2 (6.1%)	6 (5.7%)	
Mother’s height (cm), Mean ± SD	148.4 ± 4.9	148.1 ± 5.6	0.638 ^a^
Mother’s height category, *n* (%)			
<150 cm	18 (54.5%)	56 (58.9%)	0.659 ^b^
≥150 cm	15 (45.5%)	39 (41.1%)	
Monthly household income, *n* (%)			
<IDR 2,500,000	27 (81.8%)	75 (71.4%)	0.236 ^b^
≥IDR 2,500,000	6 (18.2%)	30 (28.6%)	

* Statistically significant, ^a^ unpaired *t*-test, ^b^ chi-square, ^c^ Fisher’s exact.

**Table 5:** Multivariate analysis of the factors that have a significant association with triple burden.

Characteristics	OR (95% CI)	*p*
Age (months) mean: ± SD	0.982 (0.953–1.012)	0.249
Weight-for-age		
Normal		
Underweight	0.427 (0.172–1.056)	0.066
Weight-for-length		
Normal		
Wasted	3.677 (1.165–11.607)	0.026 *

* Statistically significant.

## Data Availability

The data that support the findings of this study can be made available by the corresponding author upon reasonable request.

## Abbreviations

The following abbreviations are used in this manuscript:

ACD	Anemia chronic disease
CBC	Complete blood count
Hb	Hemoglobin
ID	Iron deficiency
IDA	Iron deficiency anemia
LBW	Low birth weight
MCH	Mean corpuscular hemoglobin
MCV	Mean corpuscular volume
MI	Mentzer index
RBC	Red blood cells
SD	Standard deviation
SF SI	Serum ferritin Serum iron
SLI	Shine and Lal index
TB	Tuberculosis
TIBC	Total iron-binding capacity
TS	Transferrin saturation
TST	Tuberculin skin test
